# Role of *Nigella sativa* and Its Constituent Thymoquinone on Chemotherapy-Induced Nephrotoxicity: Evidences from Experimental Animal Studies

**DOI:** 10.3390/nu9060625

**Published:** 2017-06-17

**Authors:** Marco Cascella, Giuseppe Palma, Antonio Barbieri, Sabrina Bimonte, Nagoth Joseph Amruthraj, Maria Rosaria Muzio, Vitale del Vecchio, Domenica Rea, Michela Falco, Antonio Luciano, Claudio Arra, Arturo Cuomo

**Affiliations:** 1 Division of Anesthesia and Pain Medicine, Istituto Nazionale Tumori—IRCCS–“Fondazione G. Pascale”, Via Mariano Semmola, 80131 Naples, Italy; m.cascella@istitutotumori.na.it (M.C.); s.bimonte@istitutotumori.na.it (S.B.); a.cuomo@istitutotumori.na.it (A.C.); 2 S.S.D. Sperimentazione Animale, Istituto Nazionale Tumori—IRCCS—“Fondazione G. Pascale”, 80131 Naples, Italy; palma.giuseppe@icloud.com (G.P.); amruthjon@gmail.com (N.J.A.); vitale_84@hotmail.it (V.d.V.); d.rea@ist iutotumori.na.it (D.R.); michelafalco_89@libero.it (M.F.); a.luciano@istitutotumori.na.it (A.L.); c.arra@istitutotumori.na.it (C.A.); 3 Clinical, Experimental and Medical Sciences, Chair of Nephrology, Department of Cardio-Vascular Medicine, University of Study of Campania “Luigi Vanvitelli”, 81100 Caserta, Italy; 4 Division of Infantile Neuropsychiatry, UOMI-Maternal and Infant Health, Asl NA 3 SUD, Torre del Greco, Via Marconi, 80059 Naples, Italy; maramuzio@yahoo.it

**Keywords:** *Nigella sativa*, Thymoquinone, natural compounds, chemotherapy-induced nephrotoxicity

## Abstract

Background: Most chemotherapeutic drugs are known to cause nephrotoxicity. Therefore, new strategies have been considered to prevent chemotherapy-induced nephrotoxicity. It is of note that *Nigella sativa* (NS), or its isolated compound Thymoquinone (TQ), has a potential role in combating chemotherapy-induced nephrotoxicity. AIM: To analyze and report the outcome of experimental animal studies on the protective effects of NS/TQ on chemotherapy-associated kidney complications. Design: Standard systematic review and narrative synthesis. Data Sources: MEDLINE, EMBASE databases were searched for relevant articles published up to March 2017. Additionally, a manual search was performed. Criteria for a study’s inclusion were: conducted in animals, systematic reviews and meta-analysis, containing data on nephroprotective effects of NS/TQ compared to a placebo or other substance. All strains and genders were included. Results: The database search yielded 71 studies, of which 12 (cisplatin-induced nephrotoxicity 8; methotrexate-induced nephrotoxicity 1; doxorubicin-induced nephrotoxicity 2; ifosfamide-induced nephrotoxicity 1) were included in this review. Conclusions: Experimental animal studies showed the protective effect of NS, or TQ, on chemotherapy-induced nephrotoxicity. These effects are caused by decreasing lipid peroxidation and increasing activity of antioxidant enzymes in renal tissue of chemotherapy-treated animals.

## 1. Introduction

Many chemotherapy drugs, such as platinum compounds (e.g., cisplatin (CP)), antimetabolite agents (e.g., methotrexate (MTX)), alkylating agents (e.g., ifosfamide (IFO)), anthracyclines (e.g., doxorubicin (DOX)) alkaloids (e.g., vincristine), antibiotics (e.g., mitomycin C and gemcitabine) and some of the newer biologics (e.g., cetuximab) are known to cause nephrotoxicity [1]. The clinical manifestations may range from an asymptomatic elevation of serum creatinine to electrolyte disturbances and acute renal failure (ARF) requiring dialysis [2]. For this reason, nephrotoxicity induced by chemotherapy could interfere with chemotherapy, since it requires a reduction in dose or discontinuation of treatment. For instance, while CP therapeutic action is dependent of dosage, the major limitation to using a high dose of this drug is the possible occurrence of strong side effects in the kidney. Indeed, in early trials, prior to the use of preventive measures (e.g., lower doses in combination with full intravenous hydration prior and after drug administration), ARF incidence resulting from CP administration was observed in more than 50% of cases [3]. Therefore, it is important to develop effective strategies in order to prevent nephrotoxicity from anti-cancer agents and investigation in this field constitutes an active area of research. Several agents, such as L-arginine [4], vitamin E alone [5] or combined with selenium [6], magnesium [7], vitamin C (combined with losartan) [8], ebselen [9], amifostine [10], theophylline [11] have been evaluated in order to investigate on their ameliorative effects on chemotherapy-induced nephropathy.

*Nigella sativa* (NS), also known as black cumin or black seed, is an annual flowering plant that belongs to the *Family ranunculaceae*. In the Middle East, North Africa and Southwest Asia, seeds of NS and other derivatives have been used in food for a long time. The chemical composition of NS is very rich, containing fixed oils (84% fatty acids) and volatile oils, amino acids, proteins, carbohydrates, alkaloids (nigellidine, nigellimine, and nigellicine), saponins, crude fiber, as well as minerals, such as calcium, iron, sodium and potassium. In addition, vitamins such as riboflavin, thiamine, pyridoxine, folic acid and niacin are present in the chemical composition. NS oil (NSO) is rich in polyunsaturated fatty acids (PUFA), such as omega-3 and omega-6 fatty acids, phytosterols and several other substances including thymoquinone (TQ) (up to 25% in volatile oil), carvacrol, t-anethole, sesquiterpenelongifolene and 4 terpinol. The principle active ingredients of NSO are TQ, dithymoquinone, thymohydroquinone and thymol (for details on the chemical composition of NS see [12]; for extraction and analytical techniques [13]).

The antiasthmatic [14], antibacterial [15], antidiabetic [16], hepatoprotective [17], antinociceptive [18], and antihypertensive [19] properties of NS and TQ have been reported in many studies. Other studies have been conducted on the effect of NS on memory, attention and cognition [20], and to investigate the anticancer activities of NS and its active compounds [21,22,23,24]. Most of these protective actions have been associated with the anti-oxidant and anti-inflammatory proprieties of TQ [25], as the free radical scavenging effect is enhanced by many factors, including the redox properties of its quinone structure, its ability to cross biological barriers and subsequent easy access to subcellular compartments [26].

Because NS and TQ have been found to be useful in combating a number of models of nephrotoxicity, such as those induced by gentamicin [27,28], or cyclosporine [29], also in a study on renal ischemia-reperfusion injury [30], there is a big interest in the role of these agents as preventive strategies for chemotherapy-induced nephropathy.

The aim of this review is to analyze and report the outcome of experimental animal studies on the protective effects of NS, and its compound TQ, against chemotherapy-associated kidney complications.

### Bibliographic Research

This systematic review was performed in agreement with Preferred Reporting Items for Systematic Reviews and Meta-Analyses (PRISMA) guidelines [31]. A computer-operated search strategy using Medline and Embase databases up to March 2017 was implemented. The search strategy for the MEDLINE database, in which used both text words and MeSH/EMTREE terms was conducted according to the following algorithm: (“thymoquinone”(Supplementary Concept) OR “thymoquinone”(All Fields)) AND (“kidney diseases”(MeSH Terms) OR (“kidney”(All Fields) AND “diseases”(All Fields)) OR “kidney diseases”(All Fields) OR “nephropathy”(All Fields))(“nigella”(MeSH Terms) OR “nigella”(All Fields)) AND nephrotoxicity(All Fields)) (“nigella”(MeSH Terms) OR “nigella”(All Fields)) AND (“kidney diseases”(MeSH Terms) OR (“kidney”(All Fields) AND “diseases”(All Fields)) OR “kidney diseases”(All Fields) OR “nephropathy”(All Fields)). For consistency, a similar search strategy was used to search the other database. In addition, a manual search of the reference lists of articles identified was also performed. Inclusion criteria for a study’s selection were as follows: conducted on animals, systematic reviews and meta-analysis on the topic containing data on nephroprotective effects of NS or its compounds compared to a placebo or any other substance. All strains and genders were included. Studies published as abstracts were excluded. Articles identified on the search were separately screened and assessed for inclusion by two reviewers (G.P., A.B.).

## 2. Results

**Figure 1:**
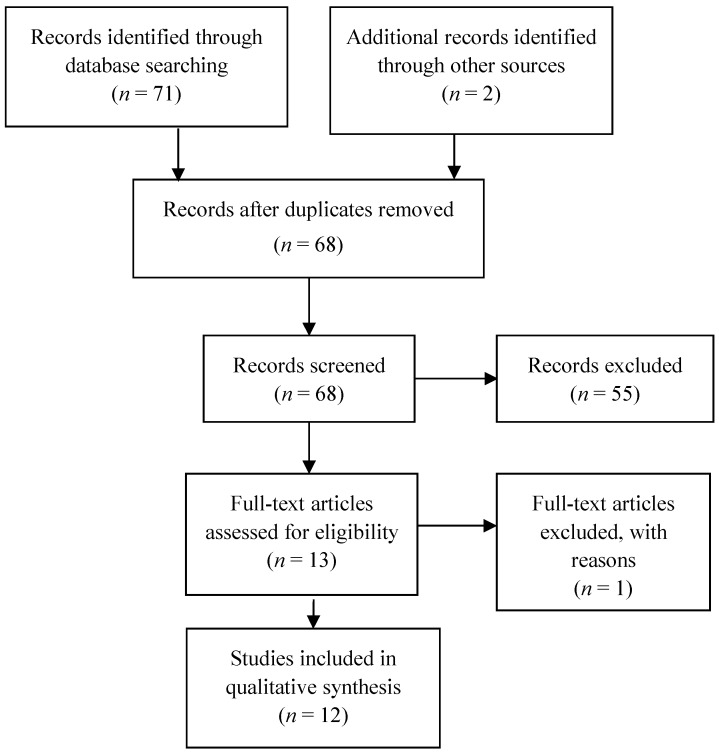
Preferred Reporting Items for Systematic Reviews and Meta-Analyses (PRISMA) Flow Diagram.

Initially, the search strategy (shown in Figure 1) identified 69 articles. From the manual search, 2 non-duplicated articles were identified and full texts retrieved. After records screening, 55 articles were excluded and 1 full-text article was excluded because, although it was included after the references search, it was not cited in the two databases used. As a result, 12 eligible studies were included in this review. Of those 12 articles, 2 were included after discussion. The narrative synthesis is presented in Table 1, whereas in Table 2 is shown a comparative analysis of the features of each study included in this review. According to the features of the available articles and to facilitate the reading of the discussion, we will outline findings in four sections: Effect of NS on cisplatin-induced nephrotoxicity (8 articles); Effect of NS on methotrexate-induced nephrotoxicity (1 article); Effect of NS on doxorubicin-induced nephrotoxicity (2 articles); Effect of NS on ifosfamide-induced nephrotoxicity (1 article).

### 2.1. Effect Of NS on Cisplatin-Induced Nephrotoxicity

Nephrotoxicity represents the principal dose-limiting adverse effect of CP [32]. The mechanism of cisplatin-induced nephrotoxicityis (CIPN) is very complex and involves the impairment of membrane transporters, such as the organic anion transporters (OATs) and the organic-cation transporters (OCTs) [33], drug excretion systems, such as the multi-drug and toxin extrusion 1 (MATE1) [34], and the multidrug resistance-associated proteins (MRP), which are membrane glycoproteins that mediate the Adenosine triphosphate (ATP)-dependent export of organic anions, including cytotoxic and antiviral drugs, from cells [35]. This cytotoxic mechanism also involves enzymes that bound to the brush border of microvillus membrane, including alkaline phosphatase (ALP), leucine aminopeptidase (LAP), γ-glutamyltransferase (γ-GT), which are collectively called renal brush border membrane enzyme (RBBME), as reported by Farooq et al. In this study, the authors showed that the activities of RBBBME changed in rat model for Ramadan fasting as a consequence of maximal velocities alterations of the enzyme reactions [36].

Mitochondrial damage and the generation of reactive oxygen species (ROS) [37], as well as a vasoconstrictor effect in the renal microvasculature, are responsible for the CP-induced renal tubular injury, which has been shown to be localized predominantly in the S3 sub segment of the proximal tubule. Increased production of lipid peroxidation biomarkers, such as malondialdehyde (MDA), 4-hydroxy-2-nonenal (4-HNE) and 8-isoprostane, is associated with the development of CP-induced nephrotoxicity (CPIN) [38]. It has been shown that acute kidney injury (AKI) (20–30%), hypomagnesemia (40–100%), hypocalcemia, hypokalemia, Fanconi syndrome, hhyponatremia (renal salt wasting), distal renal tubular acidosis, thrombotic microangiopathy, transient proteinuria, erythropoietin deficiency and chronic renal failure [39] are the renal manifestations of CP nephrotoxicity. Among the first studies performed on the protective effect of NS on CPIN there was, in 1998, the el Daly study [40], which evaluated the effect of serum enzymes activities following cysteine together with vitamin, E.; *Crocus sativus* and NS in an animal model of CPIN (3 mg/kg, intraperitoneally, i.p., for 5 alternate days in adult albino rats). In particular, the administration of NS 30 min before CP treatment partially prevented many changes in the activities of ALP, lactate dehydrogenase, malate dehydrogenase, aspartate aminotransferase and alanine aminotransferase, which were significantly decreased in CP-treated rats. Another study was conducted on the same animal model, even if with a CPIN obtained with CP (3 mg/kg, i.p.) treatment for 3 alternate days, rather than 5 days. The authors carried out histological examinations—by a pathologist unaware of details of animal groups—in addition to biochemical assays, and found mixed results. Although NS had non-significant effects on biochemical parameters, the histopathology properties of the kidneys relatively recovered after using NS [41]. These contradictory results can be explained as a possible long-term effect of NS, although the study showed several limitations, consisting of the lack of information on NS extraction procedure and especially the limited biochemical evaluation (serum creatinine, urea and triglyceride, and urine glucose). A more detailed biochemical investigation was performed by Ulu and associates [42], who evaluated the effects of TQ on lipid peroxidation—MDA and 8-isoprostane—as well as the expression of OATs, OCTs and MRPs in kidneys in CP-treated rats. They adopted an animal model of CPIN (adult male Wistar rats treated with CP 7 mg/kg i.p. in a single dose), used in other subsequent experiments, and found that MDA and 8-isoprostane levels (increased by CP administration) were reduced in rats treated with TQ (10 mg/kg in drinking water for 5 days). About the transporter systems, a significant increase in OATs and OCTs and downregulation of MRPs (MRP2 and MRP4), were observed in the combination TQ/CP treated rats compared with the CP only treated rats’ regimens. MRP transporters regulate the efflux of drugs across the brush border membrane into the urine. Moreover alternation of their expression is associated with the degree of oxidative injury in the rat kidney. This means that a decreased expression of MRP efflux transporters leads to the amelioration of CPIN, since it reduces the renal injury in rats. This study showed that the protective effect of TQ on CP nephrotoxicity may be associated with increased anion and cation transporters activity, combined with a decreased expression of MRP efflux transporters.

Interestingly, interference between the TQ administration and the abovementioned key mechanisms of CP nephrotoxicity could exist. Prior to Ulu, another study was conducted with the aim to assess the protective effect of TQ in mice and rats. TQ (4 mg/kg/day for rats and 8 mg/kg/day for mice in drinking water for 5 days before and after CP single injections) resulted in important reductions in serum urea and creatinine and significant improvement in polyuria, kidney weight, and creatinine clearance. Furthermore, renal tubules of rats treated with TQ and CP showed less degenerative damage and decreased loss of the tubular epithelium at day 5 after treatment than those treated with CP alone. Again, TQ enhanced the antitumor activity of CP in Ehrlich ascites carcinoma (EAC) bearing mice [43]. A recent investigation, in which nephrotoxicity assessment included both biochemical measurements and histological evaluations in an animal model of CPIN analogous to that of Ulu [42], has been conducted by Farooqui et al. [44]. The authors reported the effect of NSO (2 mL/kg orally) on CP-induced damage, in renal cortex and medulla separately, by the study of various serum parameters, enzymes of carbohydrate metabolism, RBBM and the antioxidant defense system. They found that NSO administration precluded CP-induced alterations in the activities of carbohydrate metabolism enzymes and in the enzymatic and non-enzymatic antioxidant parameters. Moreover, histopathology observations showed an amelioration of the CP-induced damage in the CP and NSO co-treated group. Consequently, they stated that the protective role of NSO may be the result of both mechanisms: the enhancement of the energy metabolism and the restoration of RBBM integrity [44]. This study offers evidence that NS can reverse CP-induced damages in the kidney through strong anti-oxidant effects, consisting of its ability to decrease oxidative stress by preserving the activity of the anti-oxidant enzymes [45]. Previous experimental findings proved that the same agents, such as the air pollutant diesel exhaust particle (DEP) deposited in the lungs, may complicate CPIN [46]. On the basis of these results, Ali et al. [47] conducted a preclinical study in a model of renal injury obtained with CP (6 mg/kg i.p.) combined with DEP (intra-tracheal 0.5 mg/kg). They demonstrated that treatment with TQ (orally 20 mg/kg) was able to mitigate some of the effects of CP and DEP. TQ prevented the increment of L-c-glutamyltransfrase (GGT), alanine transaminase (ALT) and aspartate transaminase (AST), although the histopathology alterations induced in lung and kidney by the CP/DEP treatment were lessened in the kidney but not in the lung. Moreover, since the IL-6 and C-reactive protein (more indicative of renal injury) concentrations were higher in the CP/DEP group than in rats treated with TQ, this investigation could suggest a more evident protective and anti-inflammatory effect of TQ on the renal damage compared with the lung. The el Daly study [40] was not the only comparative study in our search. One study was conducted to evaluate the effect of NS in comparison with Matricaria Chamomilla (MC) and Vitamin E [48]. The authors mainly investigated the neutrophil gelatinase-associated lipocalin (NAGL), a marker of renal tubular damage and early-impaired renal function [49]. They found that MC followed by NS and vitamin E improved the biochemical and pathological renal injury, as determined by increasing the body weight, normalizing the kidney functions, decreasing the oxidative stress markers, improving the apoptotic markers and minimizing the pathological changes. In another study Hosseinian et al. [50] compared vitamin E and NS, this latter used in two doses (100 and 200 mg/kg i.p.) given for 6 consecutive days. NS significantly reduced the toxic effects of CP in a dose-dependent manner in comparison with vitamin E. We included these comparative studies [40,50] because in both investigations the efficacy of NS, or TQ, was tested alone and only as a drug combination [51].

### 2.2. Effect of NS on Methotrexate-Induced Nephrotoxicity

Nephrotoxicity may represent a serious complication of MTX administration. Indeed, high dose MTX-induced renal dysfunction occurs in approximately 1.8% of patients with osteosarcoma [52]. In the literature there are not many studies conducted with the aim to evaluate the preventive effect of NS on MTX-induced nephrotoxicity. In a recent study, parameters of oxidative stress (MDA and GSH) and histopathology changes (using a 4 points grading, from 0 = normal; to 3 = severe changes) were assessed [53]. The results showed that no histopathology changes were found in all animals treated with MTX and NS, whereas the changes involved 75% of animals in the MTX group. However, although the combination MTX and NS resulted in a significant elevation in GSH level and in a reduced oxidative stress compared with the MTX group (*p* = 0.048), the biochemical evaluation did not allow firm conclusions to be drawn. In the conclusions, the authors stated that “The effect of NS on MDA levels was discarded from the analysis because of unexplained variability in the data possibly due to technical errors in measurement” [53].

### 2.3. Effect of NS on Doxorubicin-Induced Nephrotoxicity

Although the exact mechanism of DOX-induced nephrotoxicity remains unclear, it is believed that the toxicity may be mediated through mitochondrial damage with free radical formation, iron-dependent oxidative damage of biological macromolecules, LPO products, and protein oxidation [54]. This cascade induces increased glomerular capillary permeability and tubular atrophy [55]. Elsherbiny et al. [56] investigated on the effect of TQ (50 mg/kg/day oral for 3 weeks), in an animal model of doxorubin-induced nephropathy. They also examined the activity and mRNA level of nuclear factor erythroid 2-related factor 2 (Nrf2), a factor that controls the expression of several antioxidant proteins [57]. They found that treatment with TQ significantly restored renal Nrf2 mRNA and Nrf2 binding activity compared to DOX group. Thus, TQ abrogated DOX-induced renal dysfunction and tissue injury probably by enhancing cellular antioxidant capacity. Previously, Badary et al. [58] studied the influence of TQ on DOX-induced hyper-lipidemic nephropathy (DOX 6 mg/kg i.v.) in rats. The antioxidant effect of TQ in the kidney was characterized by estimating the oxidative stress as indicated by LPO formation, non-protein sulfhydryl content and catalase (CAT) activity in renal tissue. Treatment with TQ substantially reduced both hyperlipidemia and hyper-proteinuria, and restored the biomarker’s values of oxidative stress and nephrotoxicity towards normal. The results confirm the involvement of free radicals in the pathogenesis of nephropathy induced by DOX. Interestingly, TQ administered for 2 weeks after DOX showed a much less protective effect, although the author did not report the data.

### 2.4. Effect of NS on Ifosfamide-Induced Nephrotoxicity

The nitrogen mustard IFO can induce nephrotoxicity directly or by a metabolite, chloroacetaldehyde [59]. Although ifosfamide-induced nephrotoxicity manifests itself especially as proximal tubular dysfunction (Fanconi syndrome), proteinuria, impairment of glomerular filtration rate and acute renal failure can be rarely induced [60]. Progressive glutathione depletion and malondialdehyde (MAD) accumulation are the result of the kidney damage [61]. There have been few animal studies carried out assessing the influence of NS or its compounds on IFO-induced nephrotoxicity. Badary [62] demonstrated that TQ may improve the therapeutic efficacy of IFO by decreasing IFO-induced nephrotoxicity, evaluated only by biochemical measurements. However, the author evaluated the antitumor activity of TQ and IFO in mice bearing Ehrlich ascites carcinoma, EAC, tumor xenograft. Results showed that the antitumor activity of TQ + IFO was significantly higher with respect to IFO alone (73% decrease in relative tumor volume, rTV, compared to IFO alone). Moreover, a significantly reduced toxicity (as survival and body weight change, *p* < 0.05) was also detected in mice treated with TQ + IFO, compared to IFO alone.

## 3. Strengths and Limitations

The majority of animal studies on this topic have been conducted with the aim to investigate the CPIN, whereas there is a lack of studies (and a poor methodological approach) on other anticancer agents. Animal models, plant preparation and method differ among studies. About the greater effectiveness of NS or its compounds, it seems that TQ manifests the better nephroprotective effect, but a comparative study between NS and its single constituents is not available. In one study NS had no effect on renal biochemical parameters [40]. The authors given NS extract (100 mg/kg) after 2 weeks from CP, although CPIN is a quick process that begins through the apoptosis initiating reactions in the cell in the 1st h of injection [63]. Thus, differences between biochemical and histopathology results may be due to the dosage of the NS or its time-course action on biochemical parameters. Therefore, the protective factors like NS should be used in proper dose before or along with the induction of nephrotoxicity. On the other hand, previous experiments indicate that increasing TQ dose over 10 mg/kg per day was not more beneficial in protecting rats against CPIN [43]. We found a severe limitation in all studies, as allocation concealment details were not found in any among them (Table 2). This is a bias, as allocation concealment may prevent researchers from influencing which animals are assigned to each group.

## 4. Future Research Needs

A wide range of safe doses have been reported in animal studies. The LD50 of TQ was determined to be 870.9 and 104.7 mg/kg in mice and 794.3 and 57.5 mg/kg in rats in terms of oral and i.p. administration, respectively. These doses are much greater than therapeutic doses and represent the relative safety of NS and TQ [64]. In addition to these findings on NS safety, renal histopathology modifications were not observed in rats treated with NS (oral dose of 2 mL/kg) after 12 weeks of treatment [65]. Further, several studies proved that that the protective agent did not interfere with the antitumor activity of the cytotoxic agent [43,62]. All these results of effectiveness and safety shown by animal studies should encourage clinical experimentation on the topic. Moreover, a recent randomized clinical trial tested the efficacy and safety of NS oil treatment (2.5 mL, once daily orally administered) in patients with chronic kidney disease (Stage 3 and 4) due to diabetic nephropathy. Results obtained by this study, showed that in patients treated with NS (Group 2) was found a significant decrease in blood glucose (−24.90% G2 vs. −13.59% G1 = control group; *p* < 0.001), serum creatinine (−32.43% G2 vs. −9.84% G1; *p* < 0.05), blood urea (−28.50% G2 vs. −12.69% G1; *p* < 0.001), and 24 h total urinary protein levels (−45.11% G1 vs. −34.65% G2; *p* < 0.01) as well as an increment in glomerular filtration rate (+55.44% G2 vs. +13.74% G1; *p* < 0.01), 24 h total urinary volume (+28.57% G2 vs. +12.63% G1; *p* < 0.001), and hemoglobin level (+11.09% G2 vs. +9.01% G1; *p* < 0.05) compared to the control group (G1), after 12 weeks of treatments [66].

## 5. Conclusions

NS is considered an herb with great therapeutic properties for the treatment of a wide range of diseases, including cancer. Anti-cancer, anti-inflammatory properties and cancer-ameliorating effects of NS, as well as the underlying mechanisms, have largely been dissected by in vitro and in vivo studies [67].

Over the past years, significant insights into the roles of phytochemicals (as NS, quercetin, berberine, curcumin) in attenuating CPIN have been provided by pre-clinical studies. Data emerged from these studies showed that the use of renoprotective agents partially ameliorated CPIN, since not all of them were able to not interfere with the anticancer efficacy of CSP [68]. Regarding NS, experimental animal studies conducted on animal models of CPIN showed that the protective antioxidant effects of NS, or its compound TQ, are the result of both decreasing lipid peroxidation and increasing activity of antioxidant enzymes in renal tissue of chemotherapy-treated animals. However, there were some confounding factors in the animal studies reviewed. Although NS and TQ have been shown to improve kidney function in animal models of chemotherapy-induced nephrotoxicity (especially in CPIN), more experimental animal studies and ad hoc clinical studies are necessary in order to elucidate their protective effect.

## Figures and Tables

**Table 1:** Summarized results of the studies included in this review.

Animal Model	Nephrotoxicity Model	Plant Preparation and Methods	Nephrotoxicity Evaluation	Main Results	Ref.
Adult male Wistar albino rats (8–9 weeks; 240–260 g) and female Swiss albino mice (8 weeks; 21–22 g)	IFO-induced renal injury (i.p. 50 mg/kg daily for 5 days)	TQ (5 mg/kg per day) for 5 days before and during IFO treatment.	Biochemical measurements: Creatinine, Creatinine Cl, Urea, Glucosuria, Phosphaturia, GSH, GST Enzymuria	Significant amelioration of IFO-induced alteration in serum urea, creatinine, lipid peroxides and GSH in the TQ + IFO group (*p* < 0.05).	[62]
Adult male albino rats (160–180 g)	CP (i.p. 3 mg/kg for 5 alternate days)	NS extract (50 mg/kg body weight i.p.) before and after 30 min CP.	Mitochondrial oxidative phosphorylation Enzymes, Creatinine, Urea	NS reversed thetoxic effects caused by CP on Urea and creatinine serum levels. NS (given 30 min before cisplatin) partially prevented changes in the activities of serum enzymes.	[40]
Adult male albino rats (270–320 g)	CP (i.p. 3 mg/kg for 3 alternate days)	NS extract (100 mg/kg body weight, added in drinking water daily, after 2 weeks)	Serum creatinine, Urea, Triglyceride levels. Spot urine glucose. Histological evaluations.	NS had non-significant effects on biochemical parameters. The histopathology properties of the kidneys relatively recovered after using NS.	[41]
Adult male Wistar rats (180–245 g)	CP (i.p. 7 mg/kg in a single dose)	TQ (10 mg/kg in drinking water) for 5 days	Serum urea and creatinine; Lipid peroxidation (MDA and 8-isoprostane); OATs, OCTs and MRPs	Reduction of CP-induced MDA and 8-Isoprostane increments (*p* < 0.05) and significant increase in OATs and OCTs and up-regulation of MRPs in TQ + CP group	[42]
Adult male Wistar rats (150–200 g)	CP (i.p. 6 mg/kg in a single dose)	NSO (2 mL/kg orally) by gavage for 14 days prior to and 4 days following CP treatment.	Serum urea, creatinine and inorganic phosphate. Mitochondrial oxidative phosphorylation enzymes; product of LPO; RBBM and isolated RBBM vesicles. Histological evaluations.	Pre-treatment with NSO is protective from CP-induced nephrotoxicity by enhancing the energy metabolism and restoring RBBM integrity. Histopathological observations showed reduced renal injury in CP + NS group.	[44]
Adult male Wistar rats (200 g)	CP (i.p. 6 mg/kg in a single dose) + Particulate air pollution injury	TQ (20 mg/kg) orally by gavage from day 1 until 24 h before sacrifice.	Serum urea, creatinine. NAG, NGAL, IL-6, C-reactive protein, GSH, SOD, and catalase. Histological evaluations	TQ significantly abrogated many of the effects of CP and diesel exhaust particle, given alone and in combination.	[47]
Adult male Wistar Albino rats (230–300 g)	CP (i.p. 6 mg/kg in a single dose)	NS (100 and 200 mg/kg) for 6 consecutive days.	Serum urea, creatinine. Urine osmolality. Oxidative stress indices (NO, LPO)	Compared to vitamin, E.; NS significantly reduced the toxic effects of CP in a dose-dependent manner (*p* < 0.05).	[50]
Adult male Sprague-Dawley Rats (aged 26–28 weeks; weighed 250–350 g)	CP (i.p. 6 mg/kg; on the day, zeroth, 5th, 10th, 15th)	NS orally (400 mg/kg by tubing) and NS i.p. (50 mg/kg)	Serum urea, creatinine, (NAG, β-GAL), oxidative stress indices (NO, LPO), antioxidant activities (SOD), sulphur compounds (GGT, GSH), apoptotic indices (cathepsin, D.; DNA fragmentation)	M. Chamomilla + CP provided the best protection for the kidney followed by vitamin, E.; then NS orally and finally NS i.p.	[48]
Wistar Albino rats (8–9 weeks; 250–270 g). Swiss Albino mice (8 weeks; 18–20 g)	CP (i.v. 5 mg/kg in rats; i.p. 7, and 14 mg/kg in mice)	TQ (4 mg/kg/day for rats and 8 mg/kg/day for mice) in drinking water for 5 days before and after cisplatin single injections	Serum urea and creatinine, Creatinine Clearance. Histological evaluations.	TQ caused significant reductions in serum urea and creatinine and significant improvement in polyuria, kidney weight, and creatinine Clearance, as well as less tubular damage and loss.	[43]
Adult Swiss albino male mice (aged 4–6 weeks ; weighed 20–30 g)	MTX (i.p. 10 mg/kg weekly)	NSO (0.125 mL orally) for 21 days	MDA and GSH measurements on kidney homogenate. Histological evaluations	Unclear biochemical results. NS treatment resulted in preventing histopathological changes in all animals treated with NS + MTX.	[53]
Male Sprague-Dawley rats	DOX (3.5 mg/kg i.p. twice weekly for 3 weeks)	TQ (50 mg/kg/day oral for 3 weeks)	Serum urea, creatinine, albuminuria, oxidative stress indices (SOD,GST), renal inflammation (TNF-α, IL-6,IL-10); NOX-4; Nrf2. Histological evaluations	Treatment with TQ abrogated DOX-induced renal dysfunction (restored renal Nrf2 mRNA and Nrf2 binding activity) and tissue injury.	[56]
Male Wistar albino rats (180–200 g)	DOX (i.v. 6 mg/kg in a single dose)	TQ (10 mg/kg orally) 5 days prior DOX and until sacrifice	Serum urea, creatinine. Oxidative stress (LPO, non-protein sulfhydryl content, CAT).	Treatment with TQ substantially reduced both hyperlipidemia and hyperproteinuria; and restored the biomarker’s values of oxidative stress and nephrotoxicity towards normal.	[58]

Legend. CP, cisplatin; CIN, chemotherapy-induced nephropathy; NS, *Nigella sativa*; IFO, ifosfamide; i.p., intraperitoneal; TQ, thymoquinone; GSH, glutathione; GST, glutathione S-transferase; MDA, malondialdehyde; OATs, organic anion transporters; OCTs, organic cation transporters; MRPs, multidrug resistance-associated proteins; CP, cisplatin; RBBM, renal brush border membrane; NO, nitric oxide; LPO, lipid peroxidation; NSO, NS oil; MTX, methotrexate; SOD, superoxide dismutase; NAG, N-acetyl-D-glucosaminidase; NGAL, neutrophil gelatinase-associated lipocalin; DOX, doxorubicin; TNF-a, tumor necrosis factor-a; IL-6, interleukin-6; IL-10, interleukin-10; NOX-4, NADPH oxidase 4; Nrf2, nuclear factor erythroid; i.v., intravenous; CAT, catalase. Nephrotoxicity evaluation may include biochemical measurements and histological evaluations.

**Table 2:** Comparative analysis of the studies’ features included in this review.

Ref.	Number of Animals	Number of Experim. and Control Groups Report	Random.	Control Group	Details of Intervention/Exposure Group Procedures	Housing and Husbandry Conditions	Blinded Assessor for at Least One of the Outcomes Measured	* Kidney Function Tests	Hist. Changes	Statistic. Analysis
[62]	×	×	×	×	×	×		1,2,3,7		×
[40]	×	×		×	×	×		1,2,4		×
[41]	×	×		×	×	×	×	1,7	×	×
[42]	×	×	×	×	×	×		1,2,6		×
[44]	×	×		×	×	×		1,2,3,4,6	×	×
[47]	×	×	×	×	×	×	×	1,2,3,4,5	×	×
[50]	×	×	×	×	×	×		1,7		×
[48]	×	×		×	×	×		1,2,4,6	×	×
[43]	×	×	×	×	×	×		1,7		×
[53]	×	×		×	×	×	×	1,2	×	×
[56]	×	×	×	×	×			1,2,3,4,5,6,7	×	×
[58]	×	×		×	×	×		1,2,3		×

Table note: × = The criterion is satisfied; * Kidney function tests: 1 = serum urea and creatinine, or/and albumin, protein; 2 = Oxidative stress indices (e.g., nitric oxide and lipid peroxidation); 3 = Antioxidants (Superoxide dismutase activity, catalase); 4 = Sulphur compounds (Gamma glutamyltransferase); 5 = Assessment of renal inflammation (tumor necrosis factor-a; IL-6); 6 = Tissue supernatants; 7 = Urine analysis (albumin or glucose or creatinine).

## Abbreviations

CP—Cisplatin, MTX—Methotrexate, IFO—Ifosfamide, DOX—Doxorubicin, ARF—Acute Renal Failure, NS—*Nigella sativa*, NSO—*Nigella sativa* oil, PUFA—Polyunsaturated fatty acids, TQ—Thymoquinone, CIP—Cisplatin-induced nephrotoxicity, OAT—Organic anion transporter, OCT—Organic cation transporter, MATE—Multi-drug and toxin extrusion, MRP—Multidrug resistance-associated protein, ATP—Adenosine triphosphate, ALP—Alkaline phosphatase, LAP—Leucine aminopeptidase, γ-GT—γ-glutamyltransferase, RBBME—Renal brush boarder membrane enzyme, ROS—Reactive Oxygen Species, MDA—malondialdehyde, 4-HNE 4-hydroxy-2-nonenal, AKI—Acute Kidney Injury, CPIN—Cisplatin-induced nephrotoxicity, i.p.—intraperitoneal, EAC—Ehrlich ascites carcinoma, DEP—Diesel exhaust particle, GGT—L-c-glutamyltransfrase, ALT—Alanine transaminase, AST—Aspartate transaminase, NGAL—Neutrophil gelatinase-associated lipocalin, MC—Matricaria Chamomilla, Nrf2—Erythroid 2-related factor 2, CAT—Catalase.
